# Side-by-side stent deployment of novel spiral covered metal stents for malignant distal biliary obstruction with a low-confluence posterior branch

**DOI:** 10.1055/a-2888-9870

**Published:** 2026-06-26

**Authors:** Haruo Miwa, Ryo Soma, Yuto Matsuoka, Ritsuko Oishi, Yuichi Suzuki, Hiromi Tsuchiya, Shin Maeda

**Affiliations:** 1 Gastroenterological Center 26437 Yokohama City University Medical CenterYokohamaJapan; 2 Gastroenterology 218758 Yokohama City University HospitalYokohamaJapan; 3 26437 Yokohama City University Medical CenterYokohamaKanagawaJapan; 4 Department of GastroenterologyYokohama City University Graduate School of MedicineYokohamaKanagawaJapan

Covered self-expandable metal stents (SEMSs) are commonly used for unresectable malignant distal biliary obstruction; 1​ ​2​ however, they may obstruct a low-confluence posterior branch close to the biliary stricture. 3​ ​4​ The Hilzo Willow spiral covered stent (ABIS Inc., Hyogo, Japan) is a novel slim spiral covered SEMS with full inner polytetrafluoroethylene (PTFE) covering and spiral outer PTFE covering. The spiral covered structure creates gaps outside the stent lumen, which may allow bile flow from side branches and the cystic duct while maintaining the advantages of a covered SEMS, including the prevention of tumor ingrowth and removability ( Fig. 1 ).

**Fig. 1:**
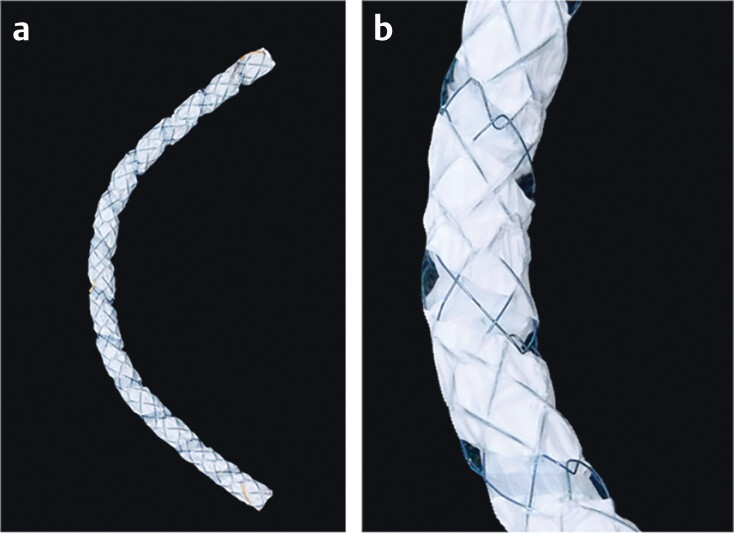
The structure of the Hilzo Willow spiral cover stent; ABIS Inc., Hyogo, Japan. ( **a** ) The overall appearance of the stent (6 mm×12 cm). ( **b** ) A magnified view showing the cross-and-hook wire structure, full inner PTFE covering, and spiral outer PTFE covering.

A 60-year-old man was referred to our hospital with obstructive jaundice due to bile duct cancer ( Fig. 2 ). Cholangiography showed a stricture in the distal bile duct with a low confluence just above the stricture. The orifice of the cystic duct was involved in the stricture. Because of the limited patency of plastic stents and the non-removability of uncovered metal stents, both options were considered unsuitable for long-term drainage. Therefore, side-by-side deployment of Hilzo Willow spiral cover stents (6 mm×12 cm) was performed. The first stent was inserted into the left hepatic duct and deployed across the papilla. Subsequently, a second identical stent was smoothly inserted alongside the first stent into the posterior branch and deployed in a side-by-side fashion ( Fig. 3 ). After deployment, bile flowed not only through the inner lumen of the stents but also through the gaps created by the spiral covered structure outside the stents ( Fig. 4 ). Computed tomography performed the following day showed the complete decompression of the gallbladder and anterior branch ( Fig. 5 and Video 1 ).

**Fig. 2:**
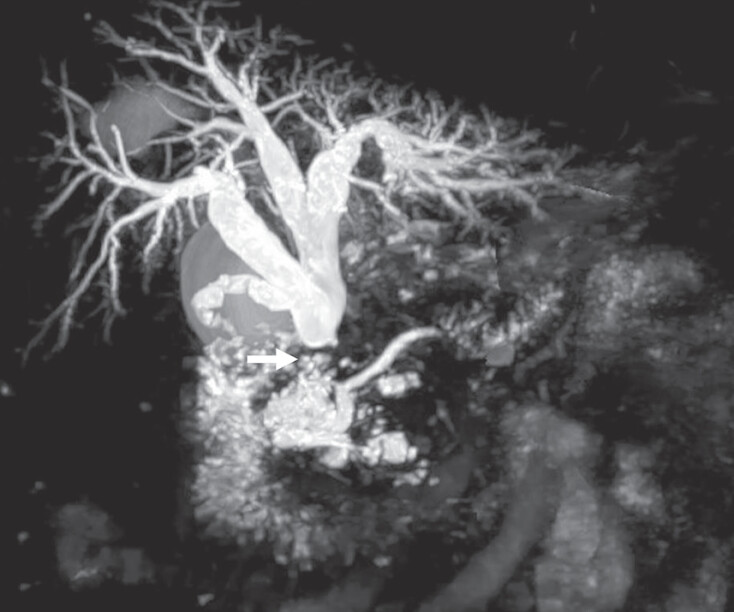
Magnetic resonance cholangiopancreatography shows the obstruction of the distal bile duct (arrow) and a low-confluence posterior branch.

**Fig. 3:**
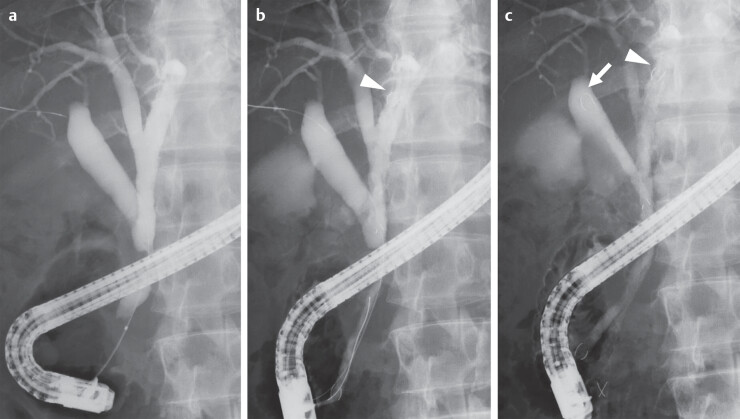
Fluoroscopic images during side-by-side stent placement. ( **a** ) Cholangiography shows the obstruction of the distal bile duct. ( **b** ) The first Hilzo Spiral Willow stent (6 mm, 12 cm) is deployed from the left hepatic duct (arrowhead) across the papilla. ( **c** ) A second identical stent is deployed from the posterior branch (arrow) across the papilla in a side-by-side fashion.

**Fig. 4:**
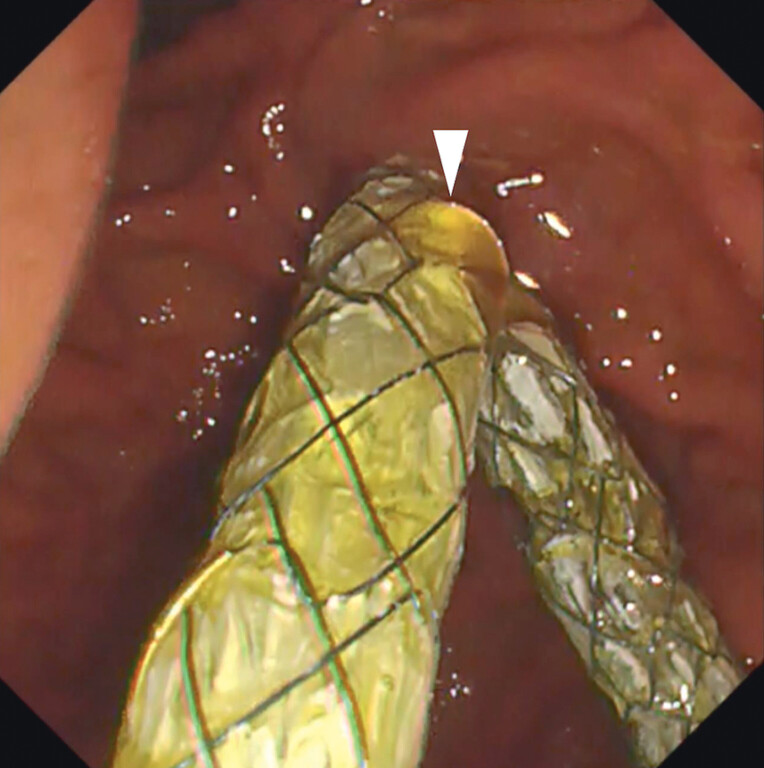
An endoscopic image after side-by-side stenting shows bile flow through the gaps created by the spiral covered structure outside of the stent lumen.

**Fig. 5:**
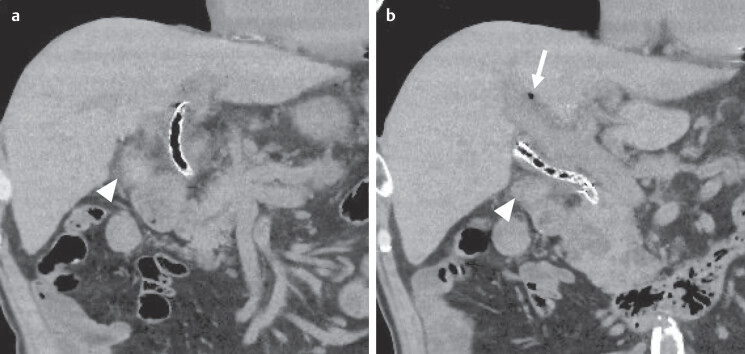
Computed tomographic images obtained the following day show the complete decompression of the gallbladder and the anterior branch, neither of which had been directly drained.

**Video 1** Side-by-side placement of the Hilzo Willow spiral cover stents for malignant distal biliary obstruction with a low-confluence posterior branch was successfully performed while preserving side-branch drainage.


To the best of our knowledge, this is the first report of side-by-side placement of a novel slim spiral covered SEMS. This technique may be a promising option for malignant hilar biliary obstruction while preserving side-branch drainage.

Endoscopy_UCTN_Code_TTT_1AR_2AZ

## Usage rights

All figures and video content, including all illustrations, schematic diagrams, and product photographs, were created entirely by the authors. The product photographs were taken by the authors using the actual device. No third-party images, icons, templates, or other copyrighted materials were used. No third party holds any rights of use for any part of the submitted figures or video.
